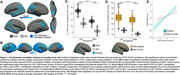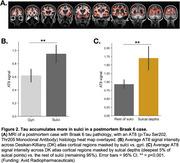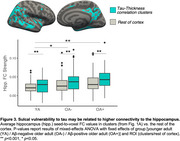# Tau pathology in Alzheimer’s disease uniquely affects sulcal depths

**DOI:** 10.1002/alz70862_110261

**Published:** 2025-12-23

**Authors:** Samira A. Maboudian, Corrina S. Fonseca, Adam Martersteck, Yishu Chao, Duygu Tosun, Lea T. Grinberg, Kevin S. Weiner, William J. Jagust

**Affiliations:** ^1^ University of California, Berkeley, Berkeley, CA USA; ^2^ University of Chicago, Chicago, IL USA; ^3^ University of California, San Francisco, San Francisco, CA USA; ^4^ Lawrence Berkeley National Laboratory, Berkeley, CA USA

## Abstract

**Background:**

Prior research suggests sulci are uniquely affected by age‐ and Alzheimer’s‐related morphological changes and Aβ pathology, but the pattern of vulnerability to tau pathology and its effects on cognition are incompletely understood. We performed this study to examine whether tau‐related atrophy preferentially affects sulci and specifically the depths, which are hypothesized to have unique connectivity patterns.

**Method:**

We used 3T MRI, amyloid and tau (FTP) PET scans from ADNI participants: 94 Aβ+ AD, 113 Aβ+ MCI, 468 cognitively normal (166 Aβ+). Correlation between cortical thickness and neocortical tau SUVR was performed using FreeSurfer’s GLM pipeline, generating an ROI of significant clusters (“clusters;” Figure 1A). We calculated overlap between the clusters and the following ROIs: whole‐cortex gyri vs. sulci, sulcal depths vs. rest of sulci. We used mixed‐effects ANOVAs to examine effects of ROI and hemisphere on the amount of overlap. We also examined the correlation between vertex‐wise cortical thickness and vertex‐wise tau, and compared the *t*‐statistic in sulcal and gyral ROIs. We then examined whether cluster volume predicts memory (ADNI‐MEM) better than the rest of the cortex using multiple linear regression. In a postmortem Braak 6 case, we calculated the amount of tau (AT8) in sulci versus gyri. Lastly, in a sample of 120 older (age 60‐94) and 64 younger adults (age 18‐33) from the Berkeley Aging Cohort Study, we examined 3T resting state connectivity strength between the hippocampus and the clusters or the rest of the cortex.

**Result:**

Tau‐thickness correlation clusters overlapped more with sulci than gyri (*p*<0.0001), and more with sulcal depths (*p*<0.0001); in the vertex‐wise comparison, sulcal *t*‐values were significantly higher than gyral (*p*<0.001; Figure 1A‐D). The volume of these clusters was more strongly associated with memory than the rest of the cortex (*p* = 0.02; Figure 1E). Tau signal in the postmortem case was higher in sulci, and in sulcal depths (Figure 2). Hippocampal connectivity strength was higher in the tau‐thickness clusters (Figure 3).

**Conclusion:**

Tau‐related thinning occurs mostly in sulci, and particularly in the depths. This sulcal vulnerability may occur because sulci accumulate more tau, potentially related to higher connectivity to the hippocampus throughout the lifecourse in these vulnerable regions.